# *RPL21* siRNA Blocks Proliferation in Pancreatic Cancer Cells by Inhibiting DNA Replication and Inducing G1 Arrest and Apoptosis

**DOI:** 10.3389/fonc.2020.01730

**Published:** 2020-09-08

**Authors:** Chaodong Li, Mei Ge, Daijie Chen, Tao Sun, Hua Jiang, Yueqing Xie, Huili Lu, Baohong Zhang, Lei Han, Junsheng Chen, Jianwei Zhu

**Affiliations:** ^1^Engineering Research Center of Cell and Therapeutic Antibody, Ministry of Education, School of Pharmacy, Shanghai Jiao Tong University, Shanghai, China; ^2^Jecho Biopharmaceuticals Co., Ltd., Tianjin, China; ^3^Shanghai Laiyi Center for Biopharmaceutical R&D, Shanghai, China; ^4^China State Institute of Pharmaceutical Industry, Shanghai, China; ^5^Jecho Laboratories, Inc., Frederick, MD, United States

**Keywords:** pancreatic cancer, RPL21, cell proliferation, cell cycle, cell apoptosis

## Abstract

**Background:**

Our previous study showed that the ribosomal protein L21 (RPL21) may play an important role in the development and survival of pancreatic cancer. In this article, RNA interference (RNAi) experiments were performed with RPL21-specific small interfering RNA (siRNA) to elucidate the mechanism by which RPL21 controls PC PANC-1 and BxPC-3 cell proliferation.

**Methods:**

In the present study, PANC-1, BxPC-3 cells, and BALB/c nude mice were used to investigate antitumor effect and mechanism by which *RPL21* controls cell proliferation and apoptosis *in vitro* and *in vivo*. The effects of *RPL21* knockdown on PANC-1 and BxPC-3 cell proliferation, cell cycle and cell apoptosis *in vitro* were determined using 3-(4,5-dimethylthiazol-2-yl)-2, 5-diphenyltetrazolium bromide assays and flow cytometry assay. The mechanism of *RPL21* regulating cell proliferation was investigated using transcriptome sequencing analysis and luciferase reporter assay. The effects of *RPL21* knockdown on PANC-1 and BxPC-3 cell proliferation *in vivo* were determined using BALB/c nude mice tumor model.

**Results:**

In PANC-1 and BxPC-3 cells, the knockdown of RPL21 expression with corresponding siRNA suppressed cell proliferation *in vitro* and *in vivo*, inhibited DNA replication, and induced arrests in the G1 phase of the cell cycle. Further results showed that the mini-chromosome maintenance (MCM) protein family (MCM2-7), CCND1 and CCNE1 were down-regulated significantly in PANC-1 and BxPC-3 cells after transfected with RPL21 siRNA, which suggests that the suppression of DNA replication is due to the reduced expression of MCM2-7 family, and the induction of G1 arrest is correlated with the inhibition of CCND1 and CCNE1. Luciferase reporter assay showed that RPL21 controls the DNA replication and G1-S phase progression possibly through the regulation of E2F1 transcription factor in PC cells. Moreover, RPL21 siRNA showed an apoptosis-inducing effect only in BxPC-3 and PANC-1 cells but not in normal HPDE6-C7 cells. The increase of caspase-8 activities and the loss of mitochondrial membrane potential after RPL21 silencing indicates that the RPL21 gene may be involved in caspase-8-related mitochondrial apoptosis.

**Conclusion:**

Our findings suggest that siRNA against the RPL21 gene possesses a potential anti-cancer activity for PC cells by inhibiting their proliferation and DNA replication, as well as inducing cell cycle G1 arrest and cell apoptosis.

## Introduction

Pancreatic cancer (PC) is exceptionally aggressive. Treatment options are limited not just the late diagnosis of the disease (1–3). Similar to other malignancies, its occurrence was a process with multi-factors and multi-steps, including activation of proto-oncogenes, inactivation of tumor-suppressor genes, alterations in cell cycle related proteins and so on. Many cancer-related genes interact with each other and are involved in the progression of PC (4). In the past decade, some proto-oncogenes (*EGFR*, *KRAS*, etc.) have been characterized as therapeutic targets for clinic treatment to improve survival rate (5). However, the current strategies haven’t demonstrated significant improvement on the survival rates yet (6). The molecular mechanism of pancreatic cancer remains unclear, and genes associated with PC progression still need to be identified.

Ribosomal proteins (RPs) have been highly conserved throughout evolution, indicating their functional importance to living organisms. In conjunction with ribosomal RNAs (rRNA) and non-ribosomal factors, RPs make up the ribosomal small (40S) and large (60S) subunits that are involved in the cellular process of translation (7). Although, initially considered to be involved only in protein synthesis, but certain RPs can mediate a variety of so called extra-ribosomal functions such as replication, transcription, RNA processing, DNA repair, and even inflammation (8, 9). In recent years, more and more RPs were found dysfunctional in tumors, such as mutation, expression levels alteration and association with differentiation and so on (10, 11). However, the exact roles of RPs in PC development are diverse and still need be further clarified.

In our previous studies, we constructed a long-term suppression model with PANC-1 cells to study the cell response to proto-oncogene *KRAS* knockdown (12). Several ribosomal protein (RPs) genes (*RPL39*, *RPL21*, etc.) were up-regulated in PC cells with long-term suppression of proto-oncogene *KRAS*. Due to the role of *KRAS* in the progression of PC, the upregulation of these RPs genes as the compensation of *KRAS* knockdown suggests that they are possibly critical for PC cells development and survival. *RPL39* has been shown to be critical for PC cell proliferation and apoptosis, whereas the apoptosis-related effect occurs only in PC cells but not in normal pancreatic duct epithelial cells (13). These results also indicated a variety of extra-ribosomal functions of *RPL39* that are independent of protein biosynthesis. Similarly to *RPL39*, *RPL21* is another up-regulated gene that encodes a ribosomal protein that is a component of the ribosomal 60S subunit and belongs to the ribosomal proteins L21E family. The extra-ribosomal functions of *RPL21* involving in PC have not been reported, and the relationship between *RPL21* and PC remains unknown.

RNA interference (RNAi) is a powerful tool to silence specific gene functions either by small interfering RNA (siRNA) or by short hairpin RNA (shRNA) (14, 15). Our previous data indicated that the expression of *RPL21* might be functionally important in PC (12). Here we tested this hypothesis by selectively reducing the *RPL21* expression using siRNA in PC PANC-1 and BxPC-3 cells. Subsequently, global changes in genes modulated in PANC-1 cells have been profiled using transcriptome analysis. The data suggest a possible functional role of *RPL21*, within a spectrum of altered gene expression, in maintaining the DNA replication and G1-S phase progression of human PC cells. Confirmation of such a scenario would allow selective therapeutic targeting of *RPL21* to modulate discrete subsets of cellular proteins that are key promoters of PC cell proliferation.

## Materials and Methods

### Cell Lines and Culture Condition

Human pancreatic cancer cell lines PANC-1 and BxPC-3 were obtained from the Committee on Type Culture Collection of Chinese Academy of Sciences (Shanghai, China). All cells were cultured *in vitro* in DMEM (Dulbecco’s modified eagle medium) high glucose medium (Gibco, Novato, CA, United States) supplemented with 10% (v/v) fetal bovine serum (FBS) (Gibco). Cells were incubated at 37°C in a humidified incubator with 5% CO_2_.

### Transfection of siRNA Targeting the *RPL21*

The gene-specific siRNAs (siL21-1 and siL21-2) against *RPL21* (Genbank NM_000982.3) was designed and synthesized by GenePharma Co., Ltd. (Shanghai, China), and the sequences were as follows: siL21-1, 5′- GCACUCUAAGAGCCGAGAUdTdT -3′ (Sense),5′-AUCUCGGCUCUUAGAGUGCdTdT-3′ (Antisense), siL21-2, 5′- GGGAAUGGGUACUGUUCAAdTdT -3′ (Sense), 5′- UUGAACAGUACCCAUUCCCdTdT -3′ (Antisense). The transfection targets of siL21-1 and siL21-2 were BLAST-searched and showed homology and similarity only to *RPL21*. The Mock-siRNA was also provided as the negative control (NC), which showed no homologous to any known human genes. The sequences of Mock-siRNA were as follows: 5′- UUCUCCG AACGUGUCACGUdTdT -3′ (Sense),5′- ACGUGACACGUUC GGAGAAdTdT -3′ (Antisense). Cells in 6-well plates (1.5 × 10^5^ cells/well) were transfected with siRNAs (40 nM) using lipofectamine 2000 reagent (Invitrogen, Carlsbad, CA, United States) according to the manufacturer’s guidelines. Usually at 72 h after transfection, gene silencing appears both at mRNA and protein levels, thus the cells were harvested and assayed at 72 h.

### Quantitative Real-Time PCR (qPCR)

Total RNAs from cells or xenograft tumor tissues were extracted using TRNzol^TM^ reagent (TIANGEN, Beijing, China) according to the manufacturer’s protocol. The concentration of total RNAs was measured using a UV spectrophotometer. First strand cDNA was synthesized from 2 μg total RNA using the First Strand cDNA Synthesis Kit (Fermentas, Glen Burnie, MD, United States). Quantitative real-time PCR (qPCR) was performed on the Mastercycler ep realplex Real-Time System (Eppendorf, Germany) using the SYBR Green qPCR kit (Fermentas, Glen Burnie, MD, United States). Gene-specific primers designed for detecting *RPL21* and *GAPDH* mRNA were shown in Supplementary Table S1. Melting curves were generated to detect primer-dimer formation and to confirm gene-specific peaks for targets. Expression data were normalized to the amount of GAPDH expressed (16).

### Western Blot Analysis

Total protein was extracted and subjected to western blotting analysis as described previously. The following antibodies were used for the western blottings: Primary polyclonal antibodies detecting E2F1, CCND1, CCNE1, MCM2, MCM3, MCM4, MCM5, MCM6, GAPDH, and MCM7 (Sangon Biotechnology, Shanghai, China). Primary monoclonal antibodies detecting RPL21 and β-Actin (Proteintech Group, Rosemont, IL, United States). After incubation with the appropriate horseradish peroxidase (HRP)-conjugated secondary antibodies for 1 h, immunoreactive proteins were visualized with Electrochemiluminescence (ECL) western blot detection reagents (Thermo Fisher Scientific, Waltham, MA, United States).

### Cell Proliferation and Colony-Forming Assay

To observe cell proliferation, cells were transfected with Mock-siRNA, siL21-1 and siL21-2 (40 nM). At 24 h after transfection, the cells were trypsinized and seeded into 96-well plates (Corning, NY, United States) at a density of 3000 cells/well in 200 μl media. The plates were incubated in a 37°C humidified incubator. On each day for 5 consecutive days, the number of viable cells was determined by 3-(4, 5-dimethylthiazol-2-yl)-2, 5-diphenyltetrazolium bromide (MTT) assay (17).

To detect colony formation, cells were transfected with Mock-siRNA (NC), siL21-1 and siL21-2 (40 nM, 24 h), and then seeded into 35-mm dishes at a density of 1000 cells/dish. The untreated cells served as the control group. Cells were fed with new growth media every 4 days. After 8 days, most of the single cells formed colony with up to more than 50 cells, and the colonies were then dyed with hematoxylin and counted.

### EdU Retention Assay

Cells were transfected with siRNAs (Mock-siRNA, siL21-1 and siL21-2) at a concentration of 40 nM for 72 h. The transfected cells were exposed to 50 μM of 5-ethynyl-2′-deoxyuridine (EdU) (RiboBio, Guangzhou, China) for 2 h at 37°C, and the cells were fixed in 4% paraformaldehyde for 30 min at room temperature, followed by the addition of 2 mg/ml glycine to neutralize the reaction. After permeabilization with 0.5% Triton-X, the cells were reacted with Apollo reaction cocktail (RiboBio, Guangzhou, China) for 30 min in the dark at room temperature. Subsequently, the DNA contents of the cells were stained with Hoechst 33342 for 30 min and visualized under a laser scanning confocal microscopy (Leica Microsystems, Buffalo Grove, IL, United States).

### Cell Cycle Analysis

Cell Cycle and Apoptosis Analysis Kit (Beyotime, Shanghai, China) was applied for cell cycle analysis. Briefly, the cells were transfected with siRNAs (Mock-siRNA, siL21-1 and siL21-2) at a concentration of 40 nM for 72 h, and then were harvested and fixed with cold 75% ethanol at 4°C overnight. The fixed cells were collected and suspended in phosphate-buffered saline (PBS) buffer containing 10 μg/ml propidium iodide (PI) and 10 μg/ml RNase A, and then incubated for 30 min at room temperature. DNA content was measured by the BD FACSCalibur (BD Biosciences, San Jose, CA, United States), and each histogram was constructed with the data from at 10,000 events. The data were analyzed and expressed as percentages of total gated cells using the Modfit LT^TM^ Software (BD Biosciences, San Jose, CA, United States).

### Transcriptome Analysis

Transcriptome sequencing and expression analysis. Human pancreatic cancer PANC-1 cells were transfected with Mock-siRNA (negative control, NC) and siL21-Mix (siL21-1 and siL21-2), respectively, at a concentration of 40 nM for 72 h. Duplicates of total RNA were prepared using TruSeq RNA Sample Prep Kit (Illumina, San Diego, CA, United States). The cDNA and cDNA library were produced, and the HiSeq 2000 Sequencing system (Illumina, San Diego, CA, United States) was used to sequence the cDNA library by Personal bio, Inc (Shanghai, China). Genes significantly regulated by siL21-Mix treatment (>2 or <2-fold change in expression) with known functions in the regulation of G1-S phase of the cell cycle and DNA replication were presented in Supplementary Table S2.

The validation of transcriptome sequencing. Gene expression profiles were validated in *RPL21* knockdown cells with qPCR assay to confirm the expression levels of *AHR*, *THBS1*, *DDIT3* and *MKNK2* (up-regulated versus the control), in addition to *E2F1*, *PCNA*, *CCND1*, *CCNE1 MCM2*, *MCM4*, *MCM5*, *MCM7*, and *KIAA0101* (down-regulated versus the control), randomly selected from differential gene after siL21-Mix (siL21-1 and siL21-2) transfection. The gene-specific primers were listed in Supplementary Table S1. All annealing temperatures were 60°C and cycling conditions as described as in section “Quantitative Real-Time PCR (qPCR).”

### Luciferase Reporter Assay

The *E2F1* expression vector pCMV-E2F1 was constructed using the pcDNA3.1 plasmid (Invitrogen, Carlsbad, CA, United States). The luciferase reporter vectors containing promoter region of genes (*E2F1*, *MCM3*, *MCM4*, *CCNE1*, *CCND1*, *MCM5*, *MCM6*, *MCM3*, and *MCM7*) were constructed using pGL-3 vector (Promega, Madison, WI, United States). The primers of full-length *E2F1* gene and promoter region of genes were presented in Supplementary Table S3. For each transfection, the cells were seeded in 6-well plates at 6 × 10^5^ cells/well. Transfection of reporter gene vectors (1 μg/well) was done using lipofectamine 2000 reagent (Invitrogen, Carlsbad, CA, United States). The pCMV-E2F1 (1 μg/well) was used as the *E2F1* expression vector to co-transfect with the reporter gene vectors, and the DNA amount was kept constant using empty pcDNA3.1 vector. Luciferase activity was measured by the Luciferase Assay System Kit (Promega, Madison, WI, United States) and TECAN Instrument (AG, Switzerland) according to the manufacturer’s protocol. Transfection experiments were done at least in triplicate. For normalization of transfection efficiency, 500 ng of Renilla reniformis luciferase expression plasmid (pRL-TK vector, Promega, Madison, WI, United States) was included in the transfection.

### Apoptosis Assay

Flow cytometry (FCM) analysis, the cells were harvested at 72 h after transfection (see transfection section of “Materials and Methods”), washed twice with ice-cold PBS and stained with Annexin V-Fluorescein isothiocyanate (FITC) and propidium iodide (PI) by Apoptosis Detection Kit (Keygen, Nanjing, China) for 10 min in the dark at room temperature. The cells with untreated cells served as the control group, and Mock-siRNA transfection served as the NC group. Then the cells were analyzed with the BD FACSCalibur and FlowJo software at 10,000 events (BD Biosciences, San Jose, CA, United States).

The Caspase-8 activities assay were analyzed using Cell Meter^TM^Caspase-8 Activity Apoptosis Assay Kit (AAT Bioquest, Sunnyvale, CA, United States) according to the manufacturer’s manual. In brief, PANC-1 and BxPC-3 cells were transfected with siRNAs (Mock-siRNA, siL21-1 and siL21-2) at a concentration of 40 nM for 72 h, then the cells were seeded into black wall, clear bottom 96-well plates and supplemented with 100 μl/well of Caspase-8 assay loading solution (containing caspase-8-specific fluorogenic substrate). Subsequently the plates were incubated with the assay loading solution for 1 h in the dark at room temperature. Then, the fluorescence intensities were measured at corresponding excitation/emission wavelength (Ex/Em = 490/525) with microplate reader (TECAN Instrument, AG, Switzerland).

Mitochondrial membrane potential assay, cell meter^TM^ JC-10 Mitochondrial Membrane Potential Assay Kit was used to measure mitochondrial membrane potential changes according to the manufacturer’s manual. The fluorescence intensities were measured at corresponding excitation/emission wavelength (Ex/Em = 500/525 nm for monomeric form of JC-10, Ex/Em = 540/590 nm for aggregating form of JC-10) with microplate reader (TECAN Instrument, AG, Switzerland). The ratios of fluorescence intensities on Em at 525/590 nm were used for data analysis.

### Tumorigenicity in Nude Mice

Eighteen BALB/c nude mice (4–6 weeks old, SPF degree, 20–24 g of body weight) were obtained from Shanghai SLAC Laboratory Animal Co., Ltd (Shanghai, China). A total of 5 × 10^6^ BxPC-3 cells were injected subcutaneously into nude mice in 0.1 ml culture medium. When the tumors reached an average volume of approximately 50 mm^3^ in about 2 weeks, the mice were randomly divided into three groups (three male and three female mice in each group), namely control, NC and siL21-Mix. All animal experiments were performed by Suzhou Xishan Zhongke Laboratory Animal Co., Ltd. (Suzhou, China) in compliance with the guidelines set by the local government^1^.

The siRNAs of injection were 2′-O-methyl (2OMe) modified to increase the stability *in vivo*. RNAi-Mate (GenePharma, Shanghai, China) was used for the *in vivo* transfection. The siRNA transfection solution containing 10 μg of siRNA was intratumorally injected at multiple sites every 3 days. The mice were closely monitored for 16 days after injection. Tumor growth was monitored by measuring the largest (a) and smallest (b) two perpendicular diameters with a caliper, and calculated the tumor volume: (v) = a × b^2^ × 0.5236.

The immunohistochemistry (IHC) procedure was performed as described by Li (18). The tumor sections were incubated at 4°C overnight with anti-human PCNA monoclonal antibody (1:100, BlueGene Biotech Co., Ltd., Shanghai, China). After washing with Tris-buffered saline (TBS), those sections were subsequently incubated for 30 min with appropriate HRP-linked secondary antibody. Immunoreactivity was developed with 3,3′-diaminobenzidine tetrahydrochloride (DAB) and CoCl_2_ enhancer tablets (Sigma-Aldrich, Shanghai, China). Finally, sections were counterstained with hematoxylin.

### Statistical Analysis

The statistical analyses were performed with SPSS 12.0 software (SPSS Inc., Chicago, United States). Results were expressed as mean values ± standard deviation (SD). The data were analyzed by Student’s *t*-test to determine statistical significance. *P* < 0.05 was considered to be statistically significant.

## Results

### Knockdown of *RPL21* Inhibits PC Cell Proliferation and Colony Formation

**FIGURE 1:**
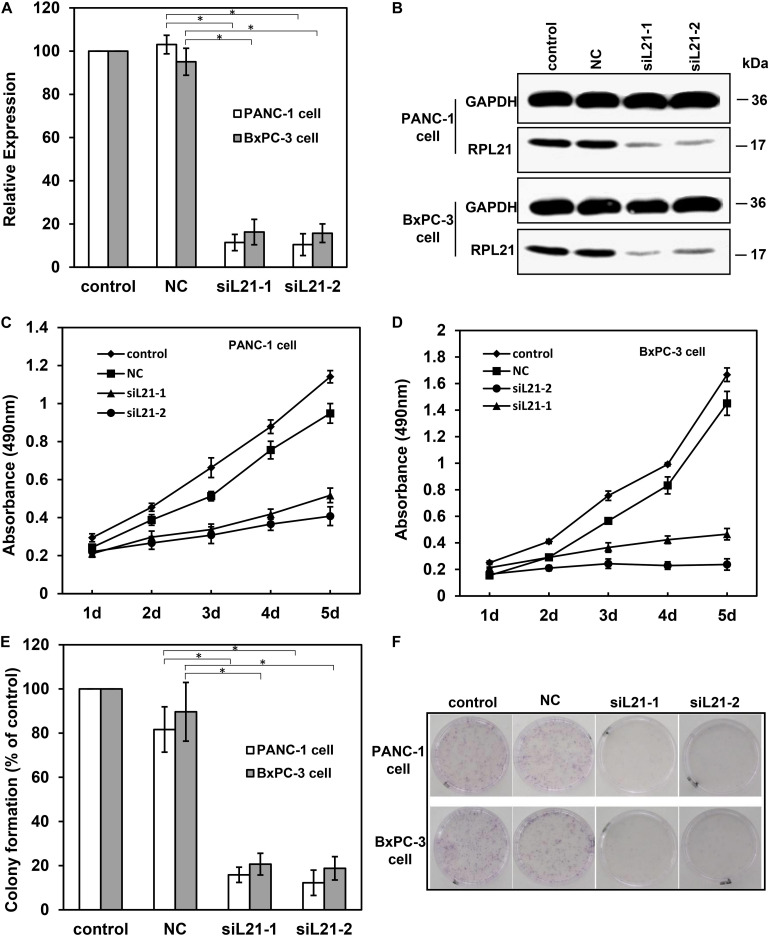
The analysis of cell proliferation on PANC-1 and BxPC-3 cells with siL21-1 and siL21-2 transfection. The quantitative real-time PCR (qPCR) assay **(A)** and the western blot assay **(B)** were used to investigate the RNAi effect of siL21-1 and siL21-2 (40 nM, 72 h) in PANC-1 and BxPC-3 cells. GAPDH and β-Actin were used as an internal control for qPCR and western blot analyses, respectively. **(C,D)** The effect of transfection with siL21-1 and siL21-2 (40 nM) on cell proliferation. The cells were detected by 3-(4, 5-dimethylthiazol-2-yl)-2, 5-diphenyltetrazolium bromide (MTT) assay on each day for 5 consecutive days. **(E,F)** For colony formation, PANC-1 and BxPC-3 cells were trypsinized and seeded into 35-mm dishes (1,000 cells/dish) at 24 h after transfection. After 8 days, cell colonies with more than 50 cells were dyed with hematoxylin and counted. The control, negative control (NC), siL21-1 and siL21-2 represented the untransfected, Mock-siRNA transfected, siL21-1 and siL21-2 transfected, respectively. Data are presented as the mean ± SD (*n* = 3). * indicates *P* < 0.05 compared to the control as determined by the Student’s *t*-test.

We designed and synthesized two gene-specific siRNAs (siL21-1 and siL21-2) for the post-transcriptional gene silencing against *RPL21* gene. As shown in Figure 1A, qPCR results revealed that, at 72 h after transfection of siL21-1 or siL21-2 (40 nM), the expression of *RPL21* was significantly decreased at the mRNA levels in PC PANC-1 and BxPC-3 cells as compared to the untreated control group (*P* < 0.05). No significant difference was observed between negative group (NC, Mock-siRNA transfection) and control groups (Figure 1A). Besides, a same trend was shown in western blot (Figure 1B) compared with qPCR assay. These results indicate that siL21-1 and siL21-2 dramatically reduce the *RPL21* expression at mRNA and protein levels in BxPC-3 and PANC-1 cells.

To determine the biological effect of silencing *RPL21* in PC BxPC-3 and PANC-1 cells, the colony formation and proliferation were measured. MTT assay was used to monitor cell proliferation for 5 consecutive days. As indicated in Figures 1C,D, the MTT analysis showed that transfection of siL21-1 and siL21-2 both inhibited cell proliferation in BxPC-3 and PANC-1 cells) as compared with the control (untransfected) and NC (Mock-siRNA transfected) groups. Consistent with MTT results, the ability of colony formation of PANC-1 and BxPC-3 cells was also decreased after transfection of siL21-1 and siL21-2 (Figure 1F). As shown in Figure 1E, the number of clones in PANC-1 transfected with siL21-1 and siL21-2 were significantly decreased by 84 and 88%, respectively, compared with control groups (untransfected) (*P* < 0.05), while in BxPC-3 cells, the inhibiting values were 79 and 81% (both *P* < 0.05). These findings indicate that ribosomal protein gene *RPL21* is closely related to proliferation of PC PANC-1 and BxPC-3 cells.

### Knockdown of *RPL21* Inhibits DNA Replication and Induces G1 Arrest in PC Cells

**FIGURE 2:**
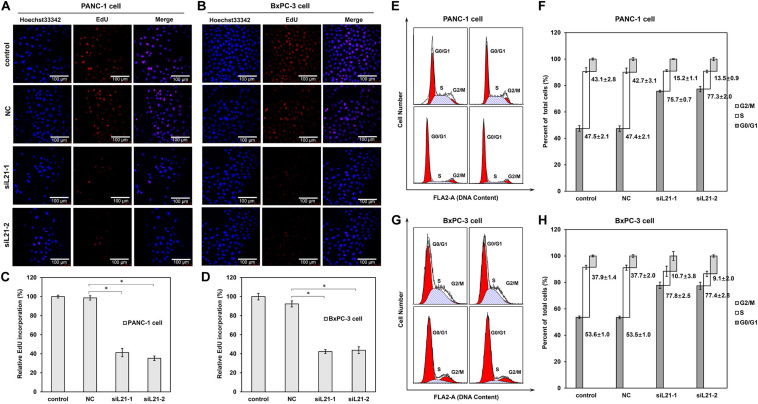
Effect of *RPL21* knockdown on DNA replication and cell cycle progression of PANC-1 and BxPC-3 cells. **(A,B)** The PANC-1 and BxPC-3 cells after transfection of siRNAs (Mock-siRNA, siL21-1 and siL21-2) (40 nM, 72 h) were incubated with 50 μM of 5-ethynyl-2′-deoxyuridine (EdU). Then the cells were stained with Hoechst 33342 for 30 min and examined by laser scanning confocal microscopy (magnification: ×400). **(C,D)** Summary graphs of the EdU retention assay for PANC-1 and BxPC-3 cells. **(E,G)** Human pancreatic cancer cells (PANC-1 and BxPC-3) were seeded in 6-well plates (2 × 10^5^ cells/well) and treated with Mock-siRNA, siL21-1 and siL21-2 (40 nM) for 72 h. Cells were harvested at the indicated time point post transfection and stained with propidium iodide (PI) for DNA cell cycle analysis. **(F,H)** Percentage of cell cycle distribution. Three independent experiments were of similar results. The untransfected cells serves as a control, and the Mock-siRNA transfected cells serves as a negative control (NC). Each bar represents the mean ± SD of triplicate analysis. * indicates *P* < 0.05 compared to the control as determined by the Student’s *t*-test.

To investigate the mechanisms underlying the altered cell proliferation, EdU incorporation assay was performed to examine the regulatory effect of *RPL21* on DNA replication. As shown in Figures 2A,B, both transfections of siL21-1 and siL21-2 drastically inhibited EdU incorporation in PANC-1 and BxPC-3 cells. The statistical analysis results showed that the EdU incorporation after transfection of siL21-1 and siL21-2 were reduced to 41% and 35% in PANC-1 cells, and to 42 and 44% in BxPC-3 cells as compared to untreated control, respectively (Figures 2C,D, all *P* < 0.05). Since the change of DNA replication tends to affect cell cycle progression (19), we were interested in whether cell cycles would be affected by the inhibition of DNA replication after *RPL21* knockdown in PC cells. Further cell cycle analysis revealed that both treatment of siL21-1 and siL21-2 (40 nM, 72 h) resulted in an accumulation in G1 phase and reduction in S phase in PANC-1 and BxPC-3 cells (Figures 2E,G). The fraction of PANC-1 cells in G0/G1 phase was increased from 47.5 to 75.7%, 47.5 to 77.3% after the treatment of siL21-1 and siL21-2, accompanied by a decrease of cells in the S (43.1 to 15.2%, 43.1 to 13.5) phase (Figure 2F) (*P* < 0.05). In BxPC-3 cells, the fraction of cells in the G0/G1 phase was increased from 37.9 to 77.8%, 37.9 to 77.4% by siL21-1 and siL21-2 treatment (*P* < 0.05), and simultaneously, we observed a decrease of cells in S (37.9 to 10.7%, 37.9 to 9.1%) phase after the addition of siL21-1 and siL21-2 (Figure 2H) (*P* < 0.05). Regarding DNA replication and cell cycle, there was no significant difference between NC (Mock-siRNA transfected) and control groups. These changes lead to a logic speculation that the inhibition of proliferation in PC PANC-1 and BxPC-3 cells may be caused by the inhibition of DNA replication and G1 arrest that resulted from silencing of *RPL21*, which suggest that *RPL21* controls proliferation and G1-S phase progression of PC cells.

### *RPL21* Controls the Expression of Important G1-S Phase and DNA Replication Regulators in PC Cells

**FIGURE 3:**
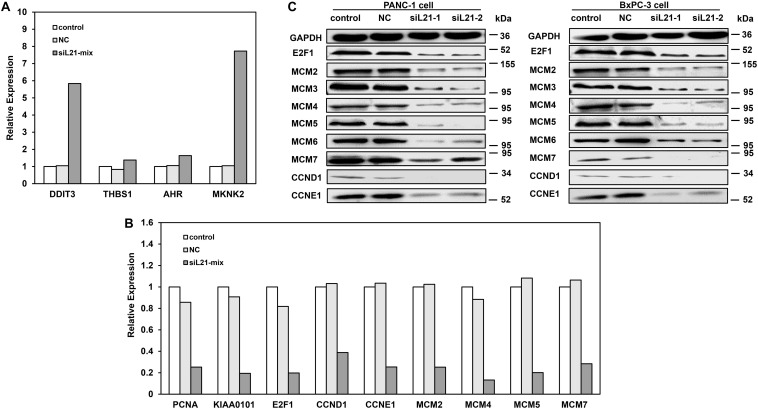
Expression analysis for genes that as the G1-S phase and DNA replication regulators after *RPL21* knockdown. **(A,B)** The validation of transcriptome analysis by quantitative real-time PCR (qPCR). Human pancreatic cancer PANC-1 cells were transfected with siL21-Mix (40 nM, 72 h), the up-regulated genes (*AHR*, *THBS1*, *DDIT3*, and *MKNK2*) in transcriptome sequencing were confirmed by qPCR. The down-regulated genes (*E2F1*, *PCNA*, *CCND1*, *CCNE1 MCM2*, *MCM4*, *MCM5*, *MCM7*, and *KIAA0101*) in transcriptome sequencing were confirmed by qPCR. **(C)** The PANC-1 and BxPC-3 cells were transfected with Mock-siRNA, siL21-1 and siL21-2 (40 nM, 72 h). The equal amounts (15 μg) of each protein sample were analyzed by western blot with antibodies of *E2F1*, *CCND1*, *CCNE1*, *MCM2*-7 and *GAPDH*. The *GAPDH* served as an internal control. The control, negative control (NC), siL21-1 and siL21-2 represented the untransfected, Mock-siRNA transfected, siL21-1 and siL21-2 transfected, respectively. Three independent experiments were of similar results.

To identify genes contributing to the observed G1 arrest and the inhibition of DNA replication after *RPL21* knockdown in PC cells, we did transcriptome sequencing analysis in PANC-1 cells. Comparing with NC groups (PANC-1 cells transfected with Mock-siRNA), 107 genes were found up-regulated and 254 genes were downregulated in siL21-Mix groups (PANC-1 cells transfected with siL21-Mix (siL21-1 and siL21-2) groups) (data was not shown). To verify the transcriptome result, qPCR assay was performed. As shown in Figures 3A,B, for up-regulated genes (*AHR*, *THBS1*, *DDIT3*, and *MKNK2*) and down-regulated genes (*E2F1*, *PCNA*, *CCND1*, *CCNE1 MCM2*, *MCM4*, *MCM5*, *MCM7*, and *KIAA0101*) that we randomly selected, the qPCR results were consistent with the transcriptome sequencing assay. Based on the roles in specific biological functions, the 361 differentially expressed genes were grouped by Gene Ontology (GO) (Supplementary Figure S1). The GO enrichment analysis showed that “cell cycle” in biological process was the most significantly modified, which is consistent with the cell cycle changes that we observed in the previous sections (Figure 2). We then focused and did further analysis on genes that are significantly regulated (<2- or >2-fold change after siL21-Mix transfection) which are known to contribute to G1-S phase progression. The significantly down-regulated genes after siL21-Mix transfection were listed in Supplementary Table S2. Western blot was used to verify the genes (as shown in Supplementary Table S2) that are involved in the regulation of G1-S progression and DNA replication. The results in Figure 3C suggest that the protein expression levels of *E2F1*, *MCM2*, *MCM3*, *MCM4*, *MCM5*, *MCM6*, *MCM7*, *CCND1*, and *CCNE1* are indeed decreased after the treatment of siL21-1 and siL21-2 in both PANC-1 and BxPC-3 cells. As the replicative helicase, the *MCM2*-*7* family is an evolutionarily conserved group of proteins that are essential for DNA replication (20). Moreover, in mammalian cells, both *CCND1* and *CCNE1* are positive regulators in G1-S progression (21). This observation suggests that, in PC cells after *RPL21* knockdown, the suppression of DNA replication is correlated with the reduced expression of *MCM2*-*7* family, and the G1 arrest is correlated with the reduced expression of *CCND1* and *CCNE1*.

### *RPL21* Regulates the Cell Cycle and DNA Replication via *E2F1* in PC Cells

**FIGURE 4:**
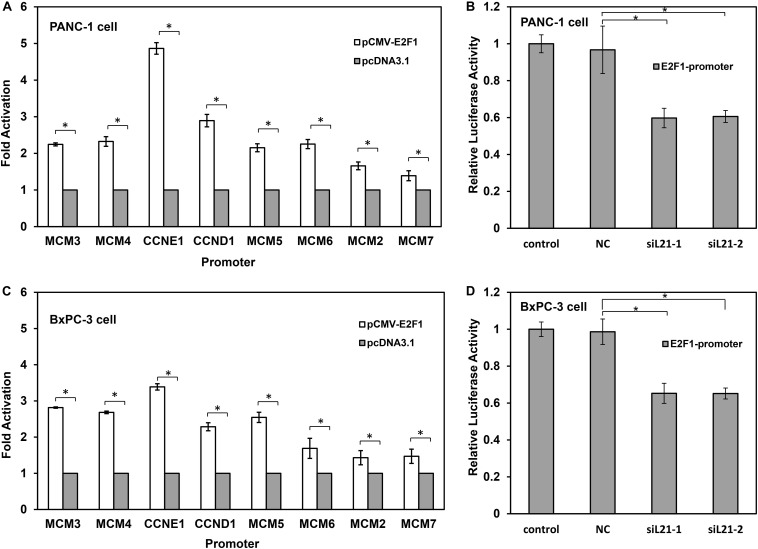
*RPL21* regulates the cell cycle and DNA replication via *E2F1* in PANC-1 and BxPC-3 cells. **(A,C)** The luciferase reporter vectors (*CCND1*, *CCNE1* and *MCM2*-*7*) were constructed using pGL-3 vectors. 1 μg reporter vector was co-transfected with 1 μg pCMV-E2F1 vector or 1 μg empty pcDNA3.1 vector (control) for each well (6-well plates) containing 6 × 10^5^ PANC-1 and BxPC-3 cells. Luciferase activity was measured 24 h after the transfection. **(B,D)** The PANC-1 and BxPC-3 cells were transfected with siRNAs with lipofectamine 2000 reagent. The control, NC, siL21-1 and siL21-2 represented the untransfected, Mock-siRNA transfected, siL21-1 and siL21-2 transfected, respectively. The cells after transfection were seeded in 6-well plates at 6 × 10^5^ cells/well, and 2 g *E2F1* luciferase reporters (E2F1-promoter) were transfected using lipofectamine 2000 reagent. Luciferase activity was measured 24 h after the transfection. Each bar represents the mean ± SD of triplicate analysis. * indicates *P* < 0.05 compared to the control as determined by the Student’s *t*-test.

The fact that *E2F1* transcription factor is positively correlated with proliferation markers suggests its effector function in G1-S phase progression in PC cells (22). *MCM2*, *MCM3*, *MCM4*, *MCM5*, *MCM6*, *MCM7*, *CCND1*, and *CCNE1* have been shown be regulated by *E2F1* (23). The results in Figure 3 showed that, the expression of *E2F1* decreased after treatment of siL21-1 and siL21-2 at both mRNA and protein levels in PC cells, which raises a possibility that the G1-S phase progression and DNA replication in PC cells may be regulated by *RPL21* via *E2F1*. To determine the effect of *E2F1* on the transcriptional regulation of *MCM2*, *MCM3*, *MCM4*, *MCM5*, *MCM6*, *MCM7*, *CCND1*, and *CCNE1* after *RPL21* inhibition, we did luciferase reporter gene assay. As shown in Figures 4A,C, whereas the *MCM2*-*7*, *CCND1*, and *CCNE1* promoter reporter vectors were activated by co-transfection of *E2F1* expression plasmid (pCMV-E2F1) in PANC-1 and BxPC-3 cells, but were not activated by co-transfection of empty pcDNA3.1 vector. Furthermore, we tested E2F1-dependent transcriptional activity in PANC-1 and BxPC-3 cells using an *E2F1* reporter gene constructs (E2F1-promoter). After siL21-1 and siL21-2 treatment, we observed down-regulation of *E2F1*-dependent transcriptional activity to 59.8 and 60.6% compared with untreated control in PANC-1 cells, and to 65.3 and 65.2% compared with untreated control in BxPC-3 cells (Figures 4B,D, all *P* < 0.05). These results show that *MCM2*, *MCM3*, *MCM4*, *MCM5*, *MCM6*, *MCM7*, *CCND1*, and *CCNE1* are targets of *E2F1* gene in PC cells, and the *RPL21* regulates *E2F1* binding to the above target genes promoter through the control of *E2F1* transcription.

### Silencing of *RPL21* Induces Apoptosis of Pancreatic Cancer Cells

**FIGURE 5:**
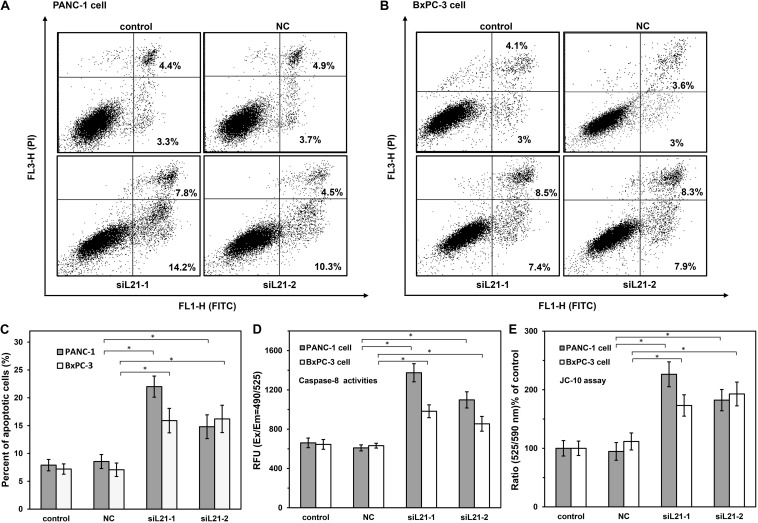
The analysis of cell apoptosis on PANC-1 and BxPC-3 cells with siL21-1 and siL21-2 transfection. **(A,B)** PANC-1 and BxPC-3 cells were transfected with *RPL21* siRNA (siL21-1 and siL21-2) and Mock-siRNA (40 nM) for 72 h, respectively, and then analyzed by Annexin V-FITC/PI staining with Flow Cytometry (FCM) analysis. The cells were analyzed with the BD FACSCalibur and FlowJo software at 10,000 events. The lower right area shows early apoptotic cells, and the upper area shows late apoptotic cells. **(C)** Summary graphs of the flow cytometry (FCM) results. **(D)** Effects of *RPL21* siRNA on Caspase-8 activities. PANC-1 and BxPC-3 cells were transfected with *RPL21* siRNA (siL21-1 and siL21-2) and Mock-siRNA (40 nM) for 72 h, respectively, then the cells were seeded into black wall/clear bottom 96-well plates (50,000 cells/well) and were added with caspase-specific fluorogenic substrate. The activities of Caspase-8 were detected at corresponding excitation/emission wavelength (Ex/Em = 490/525 nm) with microplate reader. **(E)** Effects of *RPL21* siRNA on Mitochondrial membrane potential. PANC-1 and BxPC-3 cells were transfected with *RPL21* siRNA (siL21-1 and siL21-2) and Mock-siRNA (40 nm, 72 h), respectively, then the cells were harvested and seeded into 96-well plates (60,000 cells/well) followed by the addition of JC-10 dye-loading solution. The ratio of fluorescence intensities on Em at 525/590 was used for Mitochondrial membrane potential analysis. The control, negative control (NC) and siL21-1/siL21-2 represented the untransfected, Mock-siRNA transfected and *RPL21* siRNA transfected groups, respectively. Each bar represents the mean ± SD of three separate experiments with triplicate wells per condition, *P < 0.05.

Next, we tested whether cell apoptosis would be induced by *RPL21* silencing in PC cells. Apoptosis in BxPC-3 and PANC-1 cells were measured by flow cytometry (FCM) with double-staining of Annexin V-FITC/PI. The rates of early apoptotic cells (lower right area) and late apoptotic cells (upper right area) were shown in Figures 5A,B. And quantitative data from annexin V assay was shown in Figure 5C, siL21-1 and siL21-2 significantly increased the apoptosis rate in BxPC-3 and PANC-1 cells compared to the control group (untransfected) and NC. On the contrary, siL21-1 and siL21-2 did not induce apoptosis in the normal pancreatic duct epithelial HPDE6-C7 cells, compared with control groups (untransfected) and NC (Supplementary Figure S2). These results suggest that silencing *RPL21* markedly induces apoptosis only in PC cells but not in pancreatic normal cells, which indicates that *RPL21* gene may be a therapeutic target for PC treatment.

To demonstrate the mechanism of apoptosis induced by knock down of *RPL21*, we measured the activities of Caspase-3/7, Caspase-8, and Caspase-9 by using of the Caspase-specific fluorogenic substrate. As shown in Figure 5D, compared with control (untreated) and NC (Mock-siRNA transfected) groups, the Caspase-8 (initiators of apoptosis) activities were significantly increased in siL21-1 and siL21-2 transfected groups (both in BxPC-3 and PANC-1 cells, *P* < 0.05). However, no changes were observed in the Caspase-3/7 (executioners of apoptosis) and Caspase-9 (initiator of apoptosis) activities (Supplementary Figure S3). Next, we tested the changes of mitochondrial membrane potential by JC-10 assay. JC-10 concentrates in the mitochondrial matrix where it forms red fluorescent aggregates (Ex/Em = 540/590 nm) in viable cells. However, in apoptotic and necrotic cells, JC-10 diffuses out of mitochondria. It changes to monomeric form and stains cells in green fluorescence (Ex/Em = 500/525). As shown in Figure 5E, the ratio of fluorescence intensities at Em 525/590 was significantly increased compared to control (untreated) and NC (Mock-siRNA transfected) groups (*P* < 0.05), indicating that mitochondrial membrane potential was decreased after *RPL21* silencing. These results suggested that *RPL21* gene may be involved in Caspase-8-related mitochondrial apoptosis in PC cells. Caspase-3/7 did not execute apoptosis in this type of pathway, but the AIF (Apoptosis Inducing Factor) or EndoG (Endonuclease-G) or other factors might be the executioners of apoptosis (24).

### Downregulation of *RPL21* Inhibits Tumorigenesis of PC Cells *in vivo*

**FIGURE 6:**
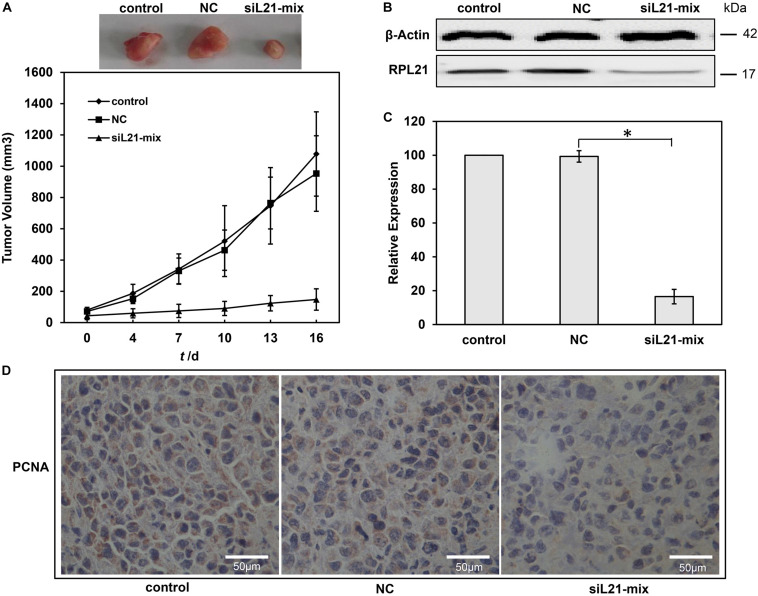
Effect of *RPL21* siRNA on pancreatic cancer growth *in vivo*. **(A)** Growth curves of BxPC-3 xenograft tumor in different treated groups. A total of 5 × 10^6^ BxPC-3 cells were injected subcutaneously into BALB/c nude mice. When the tumors reached an average volume of approximately 50 mm^3^, the mice were randomly divided into three groups (control, NC (negative control), and siL21-Mix). The siRNA transfection solution containing 10 μg of siRNA was injected every 3 days. The mice in control, NC and iL21-Mix groups were injected with transfection solution excluding siRNAs, transfection solution + Mock-siRNA and transfection solution + siL21-1 + siL21-2, respectively. Tumor growth was monitored by measuring the largest **(A)** and smallest **(B)** two perpendicular diameters with a caliper, and calculated the tumor volume: (v) = a × b^2^ × 0.5236. **(B)**The *RPL21* protein levels in different groups with western blot assays. **(C)** The *RPL21* mRNA levels in different xenograft tumor groups with quantitative real-time PCR (qPCR) assays. GAPDH and β-Actin were used as an internal control for qPCR and western blot analyses, respectively. **(D)** Tumor sections were stained with anti-human PCNA monoclonal antibody to detect proliferating cells. Each bar represents the mean ± SD of three separate experiments with triplicate wells per condition, *P < 0.05.

Finally, we determined the effect of knock down of *RPL21* on the growth of subcutaneously implanted pancreatic tumors *in vivo*. Human PC BxPC-3 cells were used to establish tumor xenografts in BALB/c nude mice. The therapeutic effect of siL21-Mix (siL21-1 and siL21-2) was evaluated by intratumorally injection. As shown in Figure 6A, the tumor volumes in the control group were 1078 ± 270 mm^3^ after 16 days treatment. On the contrary, tumors in the siL21-Mix-treated group grew significantly slower (148 ± 68 mm^3^ in volume after 16 days). The tumor volume in the NC (Mock-siRNA transfected) group was similar to that of the control group. These results suggest that injection of siL21-Mix significantly inhibited the growth of BxPC-3 tumors *in vivo*. Then, tumoral *RPL21* gene expression from tumor homogenates was evaluated by qPCR and western blot. As shown in Figures 6B,C, *RPL21* expression at both mRNA and protein levels in siL21-Mix-treated tumors was significantly decreased compared to control tumors (*P* < 0.05). Further, we examined the expression of the PCNA that is generally defined as proliferation marker in tumor xenografts. Cell proliferation in tumor section was detected by staining with anti-PCNA monoclonal antibody. As indicated in Figure 6D, there were fewer PCNA positive cells in siL21-Mix-treated tumors, suggesting that the proliferative activities of tumor cells were reduced significantly. These findings demonstrated that the knockdown of *RPL21* leads to inhibition of BxPC-3 tumors growth *in vivo*, which exhibits exceptional applicable potential of *RPL21* gene as a therapeutic target for PC.

## Discussion

Oncogene *KRAS* plays an important role in the progression of PC (5, 25). In our previous studies, we found that *RPL21* was up-regulated in two stable transfected cell lines (P-M and P-W) with long-term of low *KRAS* expression (12), which indicated that *RPL21* is possibly involved in compensation of *KRAS* silencing, and also prompted that *RPL21* is possibly critical for PC cells survival. In the present article, we confirmed this speculation, and the results showed that the knockdown of *RPL21* expression by siRNAs (siL21-1 and siL21-2) significantly inhibited the growth of human PC cells *in vitro* and *in vivo*, which suggested that *RPL21* is potentially an effective therapeutic target for PC. A pivotal issue about *RPL21* as a therapeutic target for PC is the connection between *KRAS* and the up-regulation of *RPL21*. Our previous studies showed that the up-regulation of *RPL21* gene in PANC-1 cells was because of the long-term suppression rather than the transient suppression of *KRAS* gene (12), which indicated that *RPL21* gene may interact with *KRAS* gene indirectly. In this study, siL21-1 and siL21-2 showed anti-proliferation and pro-G1 arrest functions both in RAS hyper-activation of cell lines (PC PANC-1 and BxPC-3 cells) and *RAS* normal-activation of cell lines (normal pancreatic duct epithelial HPDE6-C7 cells). However, siL21-1 and siL21-2 showed pro-apoptosis only in *RAS* hyper-activation of cell lines (PC PANC-1 and BxPC-3 cells) under the same transfection conditions (Figure 5 and Supplementary Figure S2). These results indicated that the apoptosis-inducing effect by siL21-1 and siL21-2 depends on abnormal regulation of RAS activation/deactivation. Hyper-activation of *KRAS* gene might be important for *RPL21* to regulate cell apoptosis. Our research has established the connection between *KRAS* and *RPL21* genes. Detailed relationship between the ribosomal protein and the oncogene remained unclear. Further dissecting the interaction pathway will reveal the critical signal transduction steps, which would be significantly meaningful for the development of novel treatment for PC in future.

As an integral component of the ribosomal 60S subunit of ribosome, *RPL21* primarily plays an important role in protein translation. Intuitively, by knocking down of *RPL21*, a general decline of ribosome biosynthesis would decrease the protein synthesis, including the cell proliferation-associated proteins and the cell cycle-associated proteins, which may lead to the inhibition of cell proliferation and cell cycle. However, the western blot analysis in this paper (Figure 3C) showed that the level of GAPDH appeared to be unaffected, and further GO enrichment analysis in transcriptome sequencing (Supplementary Figure S1) showed that the genes related to G1-S phase progression were significantly regulated after siL21-Mix (siL21-1 and siL21-2) transfection at mRNA levels. Both evidences suggested *RPL21* knockdown did not globally inhibit protein synthesis. Probably alternative genes may be recruited to replace deficient or defective proteins for maintaining structure-function relationships within ribosomes (26). Mammalian cells contain a large number of ribosomes. Transfection of *RPL21* siRNA only affects a portion of the ribosome pool. The unaffected ribosomes may still remain normal levels of intracellular proteins for a period of time (27).

For the first time, the present article demonstrated *RPL21*’s extra-ribosomal functions in proliferation, G1-S phase progression and apoptosis in PC cells. Interestingly, *RPL21* gene involved only in proliferation and G1-S progression regulation but not apoptosis (Supplementary Figures S2, S4, S5) in normal pancreatic duct epithelial HPDE6-C7 cells. The differential apoptosis-inducing effect of *RPL21* siRNA suggested the possibility of *RPL21* gene as a specific therapeutic target for PC treatment. In addition, the gene-specific siRNAs (siL21-1 and siL21-2) were designed to target different regions of the *RPL21*. Both siL21-1 and siL21-2 showed anti-proliferation, pro-G1 arrest and apoptosis effect in PANC-1 and BxPC-3 cells, therefore the siRNA off-target effect is unlikely in this case. This research emphasizes the important role of *RPL21* gene in PC, and highlights the need for further studies in the molecular mechanisms involved in the signaling pathway regulated through *RPL21* in PC.

**FIGURE 7:**
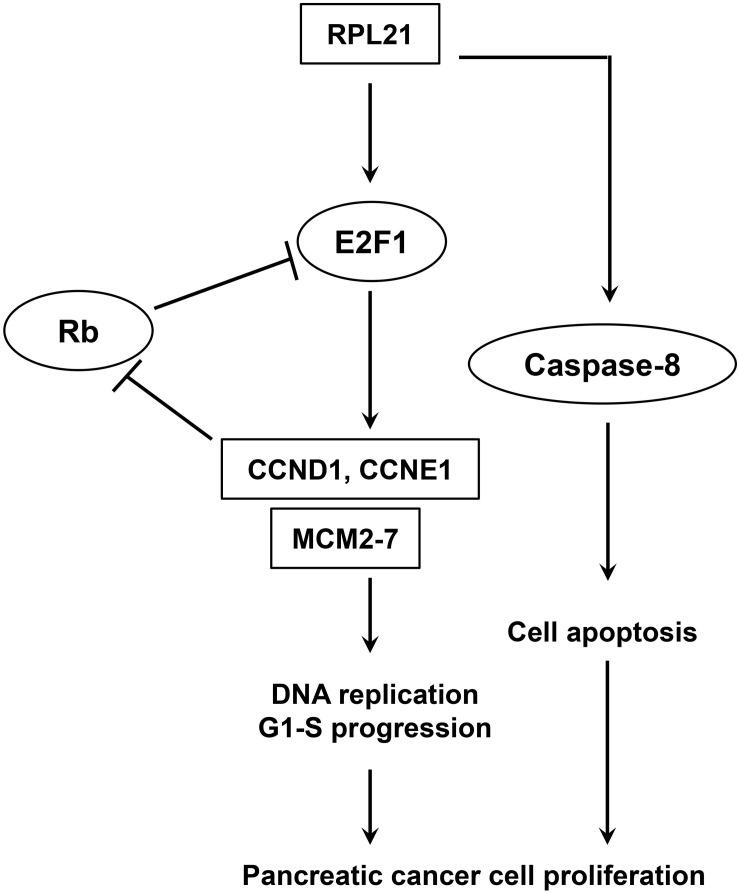
Model of *E2F1*-mediated pancreatic cancer cell proliferation.

The *E2F* family of transcription factor consists of eight members that generally associated with transcriptional activation (*E2F1*, *E2F2* and *E2F3A*) or repression (*E2F3B*, *E2F4*, *E2F5*, *E2F6*, *E2F7*, and *E2F8*) (28). They regulate important cellular responses including cell cycle progression, apoptosis, and DNA damage response (21, 29, 30), and contribute to carcinogenesis of many human tumors (31–34). The accurate transition from G1 to S phase is crucial for the control of eukaryotic cell proliferation, and its misregulation promotes oncogenesis (35, 36). The G1-S phase transition is regulated primarily by D-type cyclins (*CCND1*, *CCND2*, or *CCND3*) in complex with *CDK4*/*CDK6*, and E-type cyclins (*CCNE1*, or *CCNE2*) in complex with *CDK2* (37). During early G1, activator *E2F* proteins (*E2F1*, *E2F2*, or *E2F3A*) are bound and inhibited by retinoblastoma protein (Rb). When cells are responsive to the mitogenic signals, the *CDK4*/*CCND* and *CDK2*/*CCNE* complexes Rb, resulting in the activation of *E2F* proteins and the expression of *E2F*-responsive genes (38–40). This cluster of genes encodes cell cycle regulators required for G1/S transition (*CCNE*, *CCNA*, and *CDK1*), and components of the DNA replication machinery (*CDC6*, *ORC1*, and *MCM*2-7) (41). The *ORC/CDC6/MCM2-7* complex plays a key role for regulated helicase loading in the process of DNA replication. Errors during this process lead to cell cycle arrest at G1-S transition, and further lead to genetic disorders (42). The present paper reported that, in pancreatic cancer (PC) cells, *E2F1*, *CCND1*, *CCNE1*, and all members of the *MCM2-7* family were downregulated after inhibition of the *RPL21* expression (Figure 3C), leading to the reduction of DNA replication (Figure 2). Further luciferase reporter assay demonstrated that the knockdown of *RPL21* decreased the promoter activity of *E2F1* (Figures 4B,D). It has been confirmed that *MCM2-7*, *CCND1*, and *CCNE1* are target genes of *E2F1* transcription factor (Figures 4A,C). As shown in Figure 7, after transfection of siL21-1 and siL21-2 in PC cells, the expression of *RPL21* is downregulated, resulting in decreased transcription of the transcription factor *E2F1*. It has been shown previously that the downregulation of *CCND1* and *CCNE1* also resulted in the inactivation of *E2F1* due to the binding of non-phosphorylated Rb and *E2F1* (43), which caused the suppression of proliferation and G1-S transition through the regulation of *E2F1*. Together it is suggested that *RPL21* may control DNA replication and G1-S phase progression through *E2F1* regulation in PC cells.

The present paper revealed the role of *RPL21* on the regulation of *E2F1* for the first time. In this work, siL21-1 and siL21-2 reduce the activity of the *E2F1* promoter, but the precise molecular mechanism by which *RPL21* interacts with *E2F1* remains to be unveiled. *RPL21* protein may bind to the promoter region of *E2F1* gene to regulate its transcription. However, our luciferase reporter assay showed that *E2F1* promoter reporter gene construct was not activated by the co-transfection of *RPL21* expression vector (pCMV-RPL21) both in PANC-1 and BxPC-3 cells (Supplementary Figure S6). This result suggests that *E2F1* promoter region may not contain binding sites of *RPL21* protein. The *RPL21* protein may bind to other locations of *E2F1* gene except the promoter region to regulate the transcription of *E2F1*. In addition, *RPL21* gene may regulate the *E2F1* gene transcription indirectly. Some proteins may be used as “messenger” to regulate *RPL21*-mediated transcription of *E2F1*. Therefore, how the *RPL21* accurately regulate *E2F1* transcription will be the focus of our future research. We target to identify protein components in the signal transduction pathways that interact directly with *RPL21* that involved in the regulation of *E2F1* in our next study.

In this article, we also confirmed that *RPL21* gene was involved in Caspase-8-related mitochondrial apoptosis in PC cells. However, the transcriptome sequencing analysis in this study showed no relationship between *RPL21* gene and cell apoptosis (Supplementary Figure S1). This is probably because of that, after silencing *RPL21* in PC cells, the activities of apoptosis-related protein (such as Caspase-8) changed only at protein levels but not at mRNA levels. It is of interest to understand apoptotic pathways regulated by *RPL21* gene. In our research, the Caspase-8 activities were increased, however, the Caspase-3/7 and Caspase-9 activities remained the same after the induction of apoptosis by *RPL21* siRNA in PC cells. Under such conditions, Endo G or AIF may be released from the mitochondria as the executioner to induce cell apoptosis (24, 44). Combined with the reducing of mitochondrial membrane potential, in PANC-1 and BxPC-3 cells, *RPL21* gene may regulate the apoptosis through the Caspase-8→tBID→EndoG or Caspase-8→tBID→AIF pathway. These studies highlighted the need for further research to a more precisely defined molecular mechanism that involved in apoptotic pathway regulated by *RPL21* gene in PC cells.

In summary, the results from this study revealed a new function of the important gene *RPL21* in the biological network of PC cells for the first time. The ribosomal protein gene *RPL21* controls the PC cell proliferation and G1-S phase progression possibly through the regulation of *E2F1* transcription factor. Since little is known about the relationship between *RPL21* and human PC cells, our preliminary results provide the first information regarding the possible molecular mechanisms by which *RPL21* regulate *E2F1* transcription to control PC cell proliferation.

## Data Availability Statement

All datasets generated for this study are included in the article/Supplementary Material.

## Ethics Statement

This study was carried out in accordance with the principles of the Basel Declaration and recommendations of Guide for the Care and Use of Laboratory Animals and relevant Chinese laws and regulations. The protocol was approved by the Institutional Animal Care and Use Committee (IACUC) of Shanghai Jiao Tong University.

## Author Contributions

CL and JZ conceived and designed the experiments. CL, TS, and LH performed the experiments. CL, HL, BZ, and JC collected, analyzed, and interpreted the data. MG, DC, HJ, and YX put forward very valuable comments. CL wrote the manuscript. JZ supervised the study. All authors discussed the results and commented on the manuscript.

## Conflict of Interest

CL, LH, and JZ were employed by Jecho Biopharmaceuticals Co., Ltd. HJ, YX, and JZ were employed by Jecho Laboratories, Inc. MG was employed by Shanghai Laiyi Center for Biopharmaceutical R&D. DC was employed by China State Institute of Pharmaceutical Industry. The remaining authors declare that the research was conducted in the absence of any commercial or financial relationships that could be construed as a potential conflict of interest.

## Footnotes

1 http://xsdw.bioon.com.cn/about_12099.html
