# Supplementary material for: RPL21 siRNA Blocks Proliferation in Pancreatic Cancer Cells by Inhibiting DNA Replication and Inducing G1 Arrest and Apoptosis
Source: Front Oncol. 2020 Sep 8;10:1730. doi: 10.3389/fonc.2020.01730 (PMC7509406; doi:10.3389/fonc.2020.01730)
Supplement: Supplementary file 1 [file Data_Sheet_1.PDF]

# *RPL21* siRNA blocks proliferation in pancreatic cancer cells by inhibiting DNA replication and inducing G1 arrest and apoptosis

Chaodong Li [1,2,5], Mei Ge[1,3], Daijie Chen[1,4], Tao Sun [1,2], Hua Jiang[6], Yueqing Xie[6], Huili Lu[1,2], Baohong Zhang[1,2], Lei Han[1,2,5], Junsheng Chen[1,2], Jianwei Zhu [1,2,*]

*1 School of Pharmacy, Shanghai Jiao Tong University, Shanghai, 200240, People's Republic of China*

*2 Engineering Research Center of Cell &Therapeutic Antibody, Ministry of Education, People's Republic of China*

*3 Shanghai Laiyi Center for Biopharmaceutical R&D, Shanghai, 201203, People's Republic of China*

*4 China National Institute of Pharmaceutical Industry, Shanghai, 200040, People's Republic of China*

*5 Jecho Biopharmaceuticals Co.,Ltd., Tianjin, 300467, People's Republic of China*

*6 Jecho Laboratories, Inc., Maryland, 21704, United States*

* Correspondence author. Email: jianweiz@sjtu.edu.cn; Tel: 862134204631.

School of Pharmacy, Shanghai Jiao Tong University, 800 Dongchuan Road, Shanghai, 200240, People's Republic of China.

**Cell lines and culture condition**

The human normal pancreatic duct epithelial cell line HPDE6-C7 was purchased from the Guangzhou Jennio Biotech Co., Ltd. (Guangzhou, China). The cells were maintained in vitro in RPMI 1640 medium (Gibco, CA, USA) supplemented with 10% (v/v) fetal bovine serum (FBS) (Gibco). Cells were incubated at 37 $^{0}$C in a humidified incubator with 5% $CO_2$.

**Table S1** The gene-specific primers for quantitative real-time PCR (qPCR) assay

| Gene | Primer (Forward 5'-3') | Primer (Reverse 5'-3') |
|---|---|---|
| *AHR* | ACATCACCTACGCCAGTCGC | TCTATGCCGCTTGGAAGGAT |
| *THBS1* | TGGAACTATGGGCTTGAGAAAAC | CACTGATGCAAGCACAGAAAAGA |
| *DDIT3* | TGACCCTGCTTCTCTGGCTT | CTGGGAGGTGCTTGTGACCT |
| *MKNK2* | CCATGGTCCTGCAGAGGT | TTCTGACCAGTCCTCCAGGT |
| *RPL21* | CCACCCAGAGAAGCACACTT | TTTGTAGCCCAGAGGTCCTTTA |
| *E2F1* | ATCAAAGCCCCTCCTGAGAC | CTCAGGGCACAGGAAAACAT |
| *MCM2* | CAGTTAGTGGCAGAGCAGGTGA | TGCAGAGAGGTTGTGGATGTTG |
| *PCNA* | CTCAAGAAGGTGTTGGAGGC | GTAGGTGTCGAAGCCCTCAG |
| *MCM4* | GCCACCTCTCGTAAACGGAAA | GCACGCAGTGCTTCTTCAAA |
| *MCM5* | TGAGCACAGCATCATCAAGGAC | TAGAGAACCTTGCGCTGCATG |
| *KIAA0101* | GTTTGAACATGGTGCGGACTAA | GGCAGAGGTGGAAGAACCAA |
| *MCM7* | TGGTGGATGTGGTGGAGAAAG | TGGCAAATATCACATCTGCTGG |
| *CCND1* | CGGAGGAGAACAAACAGA | TGAGGCGGTAGTAGGACA |
| *CCNE1* | TCGATTTTGGCCATTTCTTCA | CTCCAGGAAGAGGAAGGCAA |
| *GAPDH* | GCACCGTCAAGGCTGAGAAC | GCCTTCTCCATGGTGGTGAA |

**Table S2** G1-S phase and DNA replication regulators, significantly down-regulated after siL21-Mix treatment

| Gene symbol | RefSeq Transcript ID | Gene symbol | RefSeq Transcript ID |
|---|---|---|---|
| *E2F1* | NM_005225.2 | *MCM4* | NM_005914.3 |
| *CCND1* | NM_053056.2 | *MCM5* | NM_006739.3 |
| *CCNE1* | NM_001238.2 | *MCM6* | NM_005915.5 |
| *MCM2* | NM_004526.3 | *MCM7* | NM_005916.4 |
| *MCM3* | NM_002388.4 | | |

**Table S3** The primers used in luciferase reporter assay

| Gene | Primer (Forward 5'-3') | Primer (Reverse 5'-3') |
|---|---|---|
| *RPL21*(CDS) | ATGACGAACACAAAGGGAAAGAGG | TTATGCCATGAATTCATAGGGAAT |
| *E2F1*(CDS) | ATGGCCTTGGCCGGGGCCCCT | TCAGAAATCCAGGGGGGTGAGGTC |
| *E2F1*(promoter) | CTCCTTAGGGCCACGAATTGAGGAT | TTTTACGCGCCAAATCCTTTTTGCCGCG |
| *CCND1*(promoter) | TCAGTCCCAGGGCAAATTCTAAAGG | AAACTCCCCTGTAGTCCGTGTGACG |
| *CCNE1*(promoter) | CTGTCACTTGGCCCCGCCCTGTC | GGATCCGCGCCTGCCCCCTACAC |
| *MCM2*(promoter) | ATCCGAGGTGCATCCTTCACATCCT | AGCAGTACCACGATCCTCTCCGCCA |
| *MCM3*(promoter) | AAACACTTTTTCCCCTCTTGAGC | TATGATTGGCTGAGTTCTCTGAGGT |
| *MCM4*(promoter) | GGATCAGTTGATGACCGGCCAGG | GGTTACCGTGGAAAGGCGGCCGCTT |
| *MCM5*(promoter) | CAGTGTGTTAGGGTGCGAGAACC | ACTAGCCTCACCTCTGGTTTTCCGC |
| *MCM6*(promoter) | GCCAATGTCAAATATTTCGCTGATT | ATTTGCTTAGTGCCGAGGATTCG |
| *MCM7*(promoter) | GGCGCCCATTGACTCTTTTTTTTTTCC CCA | CTTGCTCCTGGGGAAGCTGAGAATC |

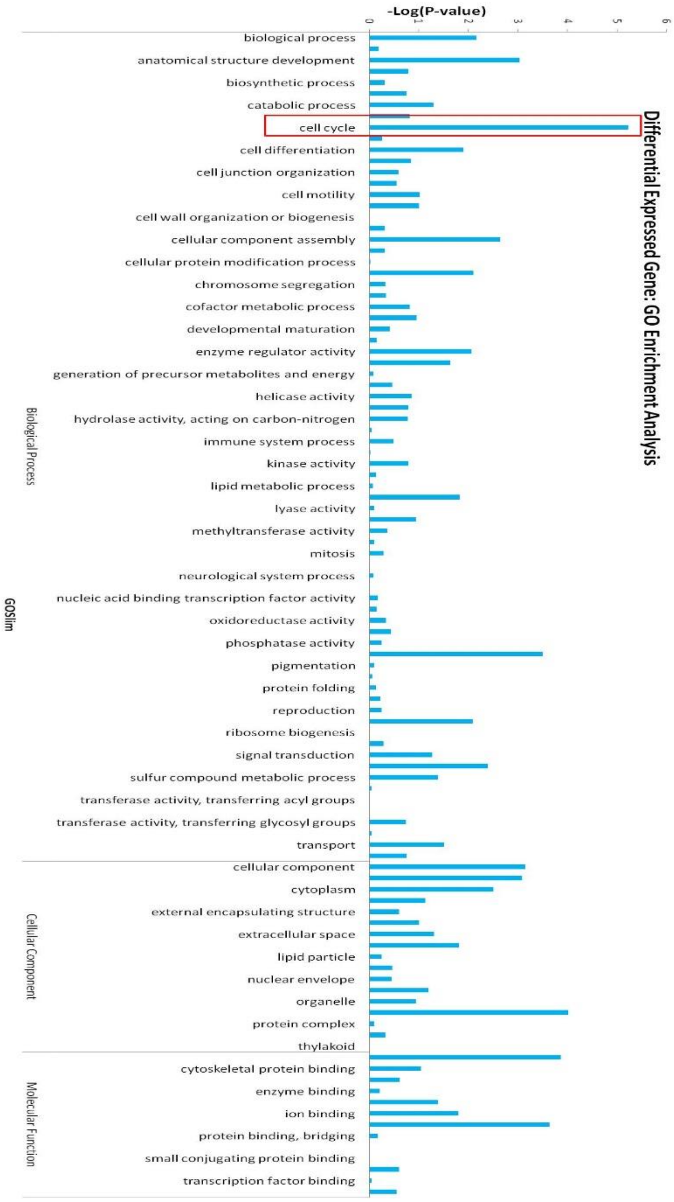


**Fig. S1** The GO enrichment analysis in transcriptome sequencing. The transcriptome sequencing analysis was implemented in PANC-1 cells. Comparing with NC groups (PANC-1 cells transfected with Mock-siRNA), 107 genes were found up-regulated and 254 genes were down-regulated in siL21-Mix groups (PANC-1 cells transfected with siL21-Mix (siL21-1 and siL21-2) groups). Based on the roles in specific biological functions, the 361 differentially expressed genes were grouped by Gene Ontology (GO).

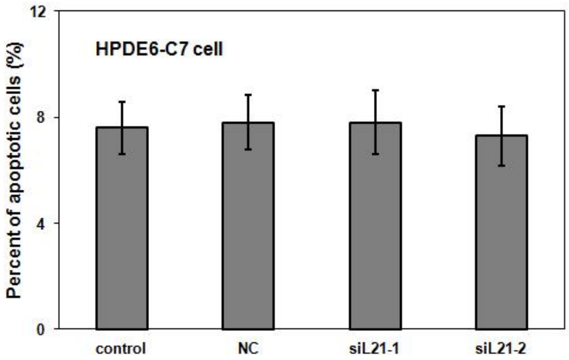


**Fig. S2** Silencing of *RPL21* with siL21-1 and siL21-2 fail to induce apoptosis of HPDE6-C7 cells. HPDE6-C7 cells were transfected with *RPL21*siRNA (siL21-1 and siL21-2) and Mock-siRNA (40 nM) for 72 h respectively, and then analyzed by Annexin V-FITC/PI staining with Flow Cytometry (FCM) analysis. The cells were analyzed with the BD FACSCalibur and FlowJo software at 10,000 events. The control, negative control (NC) and siL21-1/ siL21-2 represented the untransfected, Mock-siRNA transfected and *RPL21* siRNA transfected groups respectively. Fig. S2 is the summary graphs of the flow cytometry (FCM) results.

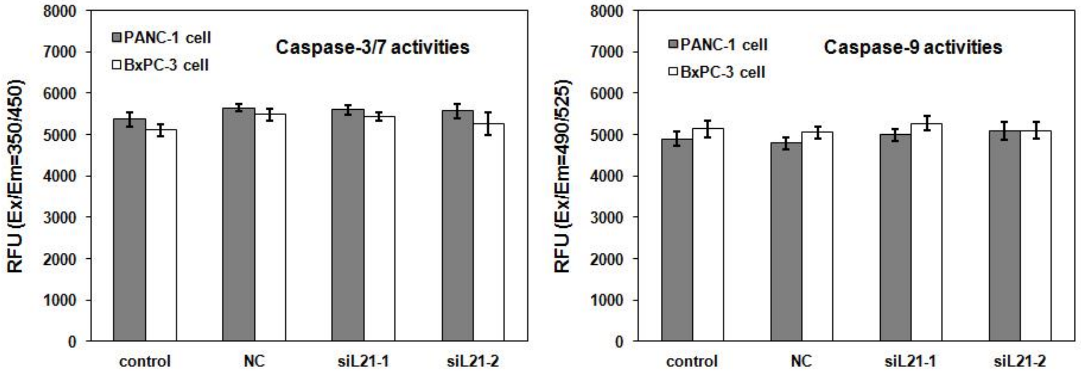


**Fig. S3** Caspase-3/7 and Caspase-9 activities are not activated by silencing of *RPL21*. PANC-1 and BxPC-3 cells were transfected with siL21-1, siL21-2 and Mock-siRNA (40 nM) for 72 h respectively, then the cells were seeded into black wall/clear bottom 96-well plates (50,000 cells/well) and were added with caspase-specific fluorogenic substrate. The activities of Caspase-3/7 and Caspase-9 were detected at corresponding excitation/emission wavelength (Ex/Em = 350/450 nm for Caspase-3/7, Ex/Em = 490/525 nm for Caspase-9) with microplate reader. The control, negative control (NC) and siL21-1/ siL21-2 represented the untransfected, Mock-siRNA transfected and *RPL21* siRNA transfected groups respectively.

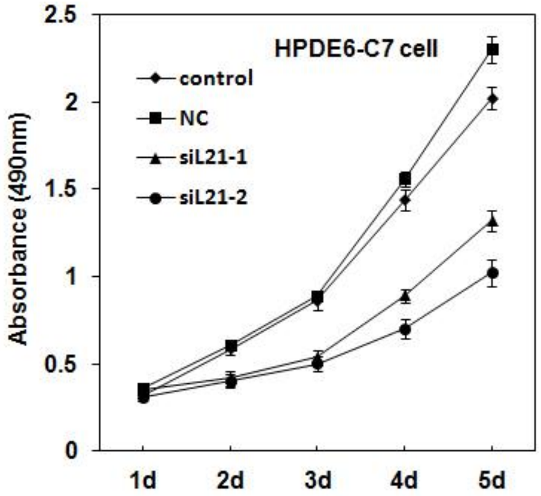


**Fig. S4** Downregulation of *RPL21* with siL21-1 and siL21-2 inhibits HPDE6-C7 cells proliferation. The effect of transfection with siL21-1 and siL21-2 (40 nM) on cell proliferation. The cells were detected by 3-(4, 5-dimethylthiazol-2-yl)-2, 5-diphenyltetrazolium bromide (MTT) assay on each day for 5 consecutive days**.** The control, negative control (NC) and siL21-1/ siL21-2 represented the untransfected, Mock-siRNA transfected and *RPL21* siRNA transfected groups respectively.

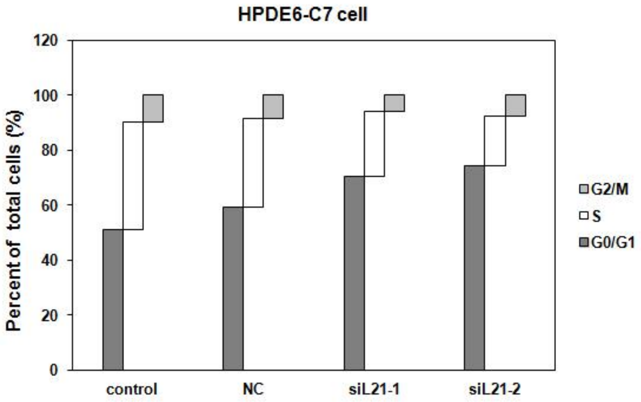


**Fig. S5** Knockdown of *RPL21* with siL21-1 and siL21-2 make G1 arrest in HPDE6-C7 cells. Human normal pancreatic duct epithelial HPDE6-C7 cells were seeded in 6-well plates ($2\times10^5$ cells/well) and treated with Mock-siRNA, siL21-1 and siL21-2 (40 nM) for 72 h. Cells were harvested at the indicated time point post transfection and stained with propidium iodide (PI) for DNA cell cycle analysis. Fig. S5 is the percentage of cell cycle distribution.

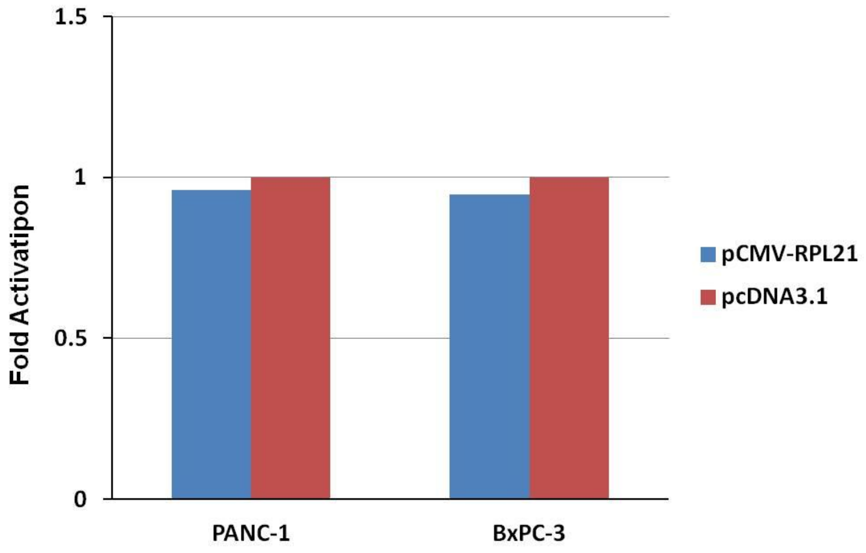


**Fig. S6** The luciferase reporter of *E2F1* promoter is not activated by cotransfection of pCMV-RPL21. The luciferase reporter vectors (*E2F1*) were constructed using pGL-3 vectors. 1 g reporter vector was cotransfected with 1 g pCMV-RPL21 vector or 1 g empty pcDNA3.1 vector (control) for each well (6-well plates) containing $6 \times 10^5$ PANC-1and BxPC-3 cells. Luciferase activity was measured 24 h after the transfection.
